# Deep incorporation of organic amendments into soils of a ‘Calardis Musqué’ vineyard: effects on greenhouse gas emissions, vine vigor, and grape quality

**DOI:** 10.3389/fpls.2023.1253458

**Published:** 2023-11-16

**Authors:** Nele Schneider, Muhammad Islam, Ralf Wehrle, Stefan Pätzold, Nicolas Brüggemann, Reinhard Töpfer, Katja Herzog

**Affiliations:** ^1^Institute for Grapevine Breeding Geilweilerhof, Julius Kühn-Institute, Siebeldingen, Germany; ^2^Institute of Bio- and Geosciences, Agrosphere (IGB-3), Forschungszentrum Jülich GmbH, Jülich, Germany; ^3^Institute of Crop Science and Resource Conservation (INRES), Soil Science and Soil Ecology, University of Bonn, Bonn, Germany

**Keywords:** *Vitis vinifera*, grapevine, plant phenotyping, greenwaste compost, biochar compost substrate, greenhouse gas emissions, climate change

## Abstract

**Background:**

Traditional wine growing regions are increasingly endangered by climatic alterations. One promising approach to mitigate advancing climate change could be an increase of soil organic matter. Here, especially subsoils are of interest as they provide higher carbon storage potential than topsoils. In this context, vineyard subsoils could be particularly suitable since they are deeply cultivated once before planting and afterwards, left at rest for several decades due to the perennial nature of grapevines.

**Methods:**

For this purpose, a biochar compost substrate and greenwaste compost were incorporated in up to 0.6 m depth before planting a new experimental vineyard with the fungus-resistant grapevine cultivar ‘Calardis Musqué’. The influence of this deep incorporation on greenhouse gas emissions and grapevine performance was evaluated and compared to a non-amended control using sensor-based analyses.

**Results:**

Increased CO_2_ emissions and lower N_2_O emissions were found for the incorporation treatments compared to the control, but these differences were not statistically significant due to high spatial variability. Only few plant traits like chlorophyll content or berry cuticle characteristics were significantly affected in some of the experimental years. Over the course of the study, annual climatic conditions had a much stronger influence on plant vigor and grape quality than the incorporated organic amendments.

**Discussion:**

In summary, organic soil amendments and their deep incorporation did not have any significant effect on greenhouse gas emissions and no measurable or only negligible effect on grapevine vigor, and grape quality parameters. Thus, according to our study the deposition of organic amendments in vineyard subsoils seems to be an option for viticulture to contribute to carbon storage in soils in order to mitigate climate change.

## 1 Introduction

Viticulture is shaped by the interplay of grapevine cultivar, location, soil, and climatic conditions, with the latter being of upmost importance, since they influence, for instance, soil moisture, grapevine development as well as quantity and quality of produced wines (Duchêne et al., 2010; Berg and Sheffield, 2018; van Leeuwen et al., 2018). As climate change progresses, drastic changes in annual temperature as well as rainfall are to be expected in future, which will presumably endanger the suitability of traditional wine growing regions (Hannah et al., 2013). One promising strategy to face the challenges of climate change that has lately gained increasing attention is removing greenhouse gases (GHG) from the atmosphere in form of carbon (C) sequestration in soils (Lal, 2004; Powlson et al., 2011). Soils represent the largest terrestrial C store and, thus, offer a major opportunity as long-term C sink (Batjes, 1996; Lal, 2004). European vineyards exhibit among the lowest soil C contents and soil fertility in agricultural systems due to frequent tillage, erosion, or soil compaction (Gerzabek et al., 2005; Coulouma et al., 2006; Angers et al., 2011; Napoli et al., 2017). Therefore, vineyard soils offer a promising potential to store C. Brunori et al. (2016), for instance, showed that solely through a proper management vineyards can act as C sinks. Soil organic carbon (SOC) content could further be increased by the incorporation of different organic amendments (OA) (Morlat and Chaussod, 2008; Calleja-Cervantes et al., 2015; Marín-Martínez et al., 2021). However, SOC content mirrors C input on the one hand and organic matter degradation on the other hand with the latter depending, among other factors, on OA quality (Kögel-Knabner, 2017). Here, biochar-based strategies have been proposed as negative emission technologies for climate change mitigation in the special report of the Intergovernmental Panel on Climate Change (IPCC) (2018), because biochar consists of stable C compounds that are highly resilient to degradation processes and, thus, offers the potential to be stored more or less permanently in agricultural soils (Lehmann, 2007). However, several studies suggested combining biochar and compost in order to gain synergistic effects of both materials for soils as well as plants (Fischer and Glaser, 2012; Hua et al., 2012), with the latter being a widely studied organic material used in viticulture (Mugnai et al., 2012; Schmidt et al., 2014). Thus, we included both a compost and a biochar-compost treatment in this study.

Incorporation of OA can positively affect biological, chemical, and physical soil properties such as microbial diversity and activity, bulk density, water holding capacity, cation exchange capacity, soil aeration, and nutrient retention (Morlat and Chaussod, 2008; Powlson et al., 2011; Giagnoni et al., 2019; Blanco-Canqui, 2021). In general, soil is known to substantially influence grapevine development and, consequently, grape and wine quality (White et al., 2007; van Leeuwen et al., 2018). Therefore, it is of high importance to evaluate the effects of modified soil properties due to OA incorporation on grapevine performance. So far, results of different studies are contradictory. While beneficial effects of OA incorporation on parameters such as vegetative growth or yield could be shown in some studies (Genesio et al., 2015; Gaiotti et al., 2017; Tangolar et al., 2020; Marín-Martínez et al., 2021; Wilson et al., 2021), others reported no or only negligible influence on grapevine performance (Mugnai et al., 2012; Schmidt et al., 2014; Baldi et al., 2022). Some studies reported even negative effects on grape quality parameters (e.g., Gaiotti et al., 2017; Botelho et al., 2021).

In most studies, OA were incorporated in the topsoil of established vineyards and, in consequence, subjected to mineralization processes due to aeration or microbial activity. This in turn might lead to enhanced emissions of potent GHG such as carbon dioxide (CO_2_), methane (CH_4_), or nitrous oxide (N_2_O) that could further exacerbate climate change (Thangarajan et al., 2013; Longbottom and Petrie, 2015; Brenzinger et al., 2018; Marín-Martínez et al., 2021). Subsoils, on the other hand, provide a large capacity for C sequestration by physical isolation of C, thereby, reducing the risk of mineralization, i.e., degradation (Powlson et al., 2011). Morlat and Chaussod (2008) showed in their long-term study that changes in vineyard subsoils first occur after several years up to decades when OA are incorporated annually in topsoils. One reasonable alternative could therefore be the incorporation of OA directly in the subsoil (Powlson et al., 2011; Chenu et al., 2019). As vineyard soils are cultivated deeply once before planting and are then left at rest for several decades (Coulouma et al., 2006), we assume that these subsoils offer great potential for long-term C storage. This is why in this work two OA – biochar compost substrate and greenwaste compost – were incorporated in the subsoil of an experimental vineyard before it was planted.

On the other hand, deep incorporation of organic materials may lead to priming effects in SOC mineralization or to enhanced GHG emissions, for instance, of N_2_O, as a consequence of low redox potential in subsoils. Hence, effects of such a deep incorporation on GHG emissions and plant development need to be assessed thoroughly in order to consider multiple consequences before formulating application recommendations for viticulture. In this respect, sensor-based methods are beneficial since they allow for rapid, precise and objective data acquisition as well as analysis (Li et al., 2014). Currently, a multitude of sensors is available to assess soil parameters, measure gas fluxes, and describe different plant traits (Li et al., 2014; Oertel et al., 2016; Wehrle et al., 2021). Leaf chlorophyll or nitrogen content, for instance, can easily be determined with simple, universally applicable hand-held devices (Cerovic et al., 2012; Cerovic et al., 2015) and 3D scanners can be implemented, for example, to gain information about grape bunch architecture (Rist et al., 2018). Further, sensors that are more specialized allow for the characterization of skin and cuticle of grapevine berries, for instance, which are important barriers towards pests and diseases (Letaief et al., 2008; Herzog et al., 2015; Herzog et al., 2022).

In order to gain a holistic overview of the proposed deep OA incorporation approach, the aims of this study were: (1) analyzing GHG emissions from the soil in order to assess degradation of OA and (2) measuring the effects of this deep incorporation on grapevine performance and grape quality parameters using suitable sensor-based analyses.

## 2 Materials and methods

### 2.1 Study site, climate, and soil

The experimental vineyard was planted in 2018 with the downy and powdery mildew -resistant cultivar ‘Calardis Musqué’ (DEU098_VIVC4549_DEU098-2018-062) grafted onto rootstock SO4 (Selektion Oppenheim 4, VIVC11473). The experimental vineyard is located at JKI Geilweilerhof in Siebeldingen, Germany (49°21.7470 N, 8°04.6780 E). It consisted of 12 rows oriented in north-south direction with an inter-row spacing of 2 m and grapevine spacing of 1 m. Grapevines were trained in a vertically shoot positioned trellis system without irrigation. Every second vine row was covered with natural spontaneous grass and the alternating rows were mostly kept free of vegetation by superficial soil tilling.

‘Calardis Musqué’ [Bacchus (VIVC851) x Seyval (VIVC11558)] provides resistances against *Botrytis* bunch rot (*Botrytis cinerea* Pers.), powdery mildew (*Erysiphe necator* (Schwein.) Burrill), and downy mildew (*Plasmopara viticola* [Berk. & Curt.] Berl. & De Toni) as well as against black rot (*Guignardia bidwellii* (Ellis) Viala and Ravaz). Consequently, in average 60% of regular plant protection applications were conducted, i.e. 60% of the recommended application rate for susceptible grapevine cultivars.

The climate in this region was recorded by a weather station (Lambrecht Meteo GmbH, Göttingen, Germany) located in the fields of JKI Geilweilerhof (approx. 600 m from the experimental vineyard).

**Figure 1:**
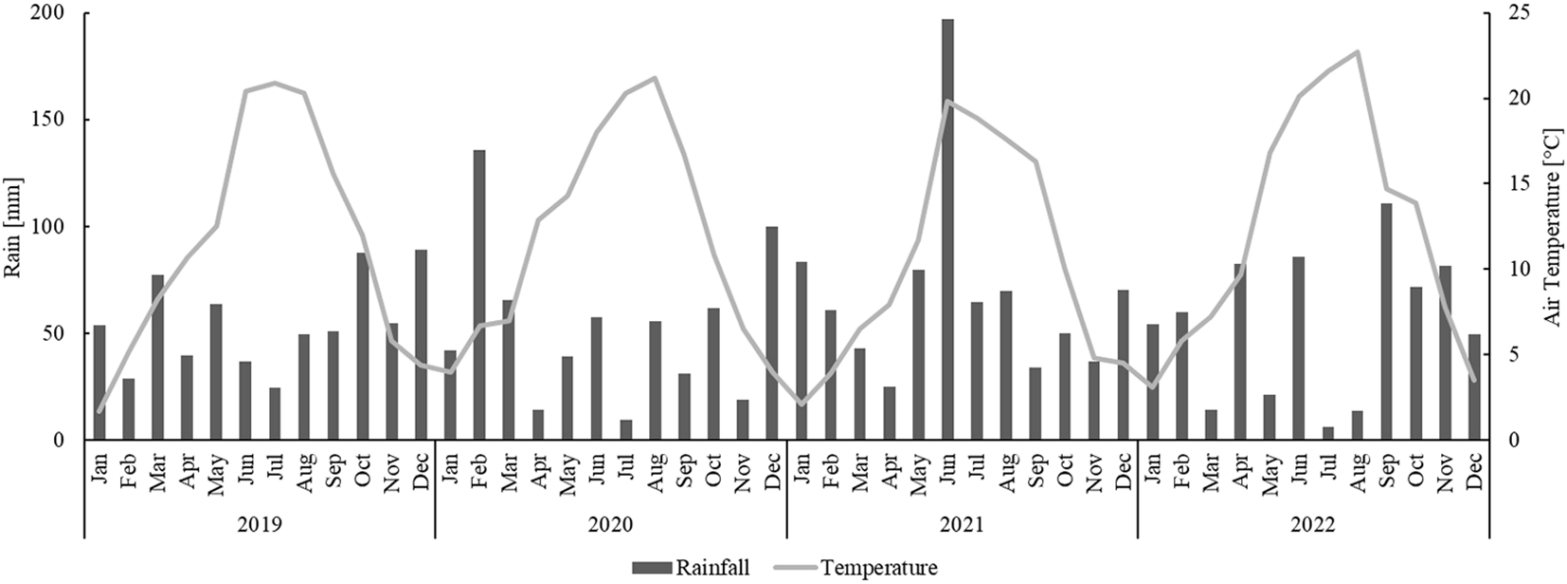
Monthly total rainfall and average air temperature for the years 2019-2022 obtained from the Siebeldingen meteorological station.

**Figure 1** shows total rainfall and mean temperature for each month of the four experimental years (2019-2022). Clear differences can be observed between the years especially in monthly rainfall during the growing period.

The plot was exposed to the south with a slope of approx. 7°. Despite the small plot length of 93 m, the soils revealed considerable heterogeneity along the slope. Soils were formed from Mesozoic sedimentary rock and had silty-loamy to clayey texture. Prior to organic amendment incorporation, soil organic carbon content in the topsoil of three reference profiles at the edge of the experiment amounted to 9 – 20 g kg^-1^. The plot had originally been used as a vineyard for decades but lied fallow several years before the experiment was established. Therefore, the soils had been ploughed or spaded to approx. 0.6 m depth in earlier times as is common practice in most German wine growing regions. This is why the natural soil horizontation was somehow disturbed. In addition, up to 0.7 m of allochthonous loess was applied in the uppermost part of the plot in order to level out the lateral slope. According to the World Reference Base of Soil Resources (WRB-FAO, 2015) the soils referred to Terric Anthrosols (top slope), Chromic Vertisols (middle slope), and Vertic Cambisols (foot slope).

### 2.2 Organic amendment incorporation and classification

In 2018, two organic amendments (OA) with a wide C:N ratio were incorporated into the subsoil before planting the vineyard. A randomized strip design was set up in parallel to the later planted vine rows with two repetitions leading to four rows of 93 grapevines per treatment. The OA were distributed by superficial mixing with a chisel, followed by deep incorporation to 0.6 m depth with a tractor-driven spader. Further, a control treated accordingly but without applying OA was utilized. In total, three experimental treatments were tested:

- Biochar compost substrate (BCS) – application rate of 30 t ha^-1^ (fresh matter)
- Greenwaste compost (GC) – application rate of 50 t ha^-1^ (fresh matter)
- Control (Co) – no incorporation of organic amendments

The application rates of BCS and GC were normalized on the total organic carbon (TOC) rates for each treatment. This resulted in application rates of 30 t ha^-1^ BCS and 50 t ha^-1^ GC with 10.5 t ha^-1^ TOC for both treatments. These amounts represent the maximum legally approved application rate to be in line with the German Fertilization Ordinance (“Düngeverordnung - DüV”; Bundesministerium der Justiz und für Verbraucherschutz und Bundesamt für Justiz, 2017). The commercial BCS product was provided by Palaterra GmbH (Hengstbacherhof, Germany) and GC by Zeller Recycling GmbH (Mutterstadt, Germany). Laboratory parameters for the OA can be seen in **Table 1**. Here, TOC and total nitrogen (TN) were determined via dry combustion and elemental analysis (ISO 10694) by two repeated measurements (Wehrle et al., 2021). If present, inorganic carbon was determined by gas volumetric Scheibler method (ISO 10693). Otherwise, if no inorganic carbon was present, total carbon was rated as TOC for further analyses. Plant available phosphate (P_avl_) and potassium (K_avl_) were determined by the OA provider (pers. communication) according to CAL method (Schüller, 1969).

**Table 1:** Composition of the applied biochar compost substrate (BCS) and greenwaste compost (GC).

	TOC	TN	C:N ratio	P_avl_	K_avl_
**BCS**	256	5.33	48.0	1.58	5.18
**GC**	213	7.97	26.7	1.69	3.38

TOC, total organic carbon [g kg^-1^]; TN, total nitrogen [g kg^-1^]; P_avl_, plant available phosphate [g kg^-1^]; K_avl_, plant available potassium [g kg^-1^].

### 2.3 Greenhouse gas measurements

A dynamic closed chamber-based survey analysis was performed to measure soil-atmosphere exchange of CO_2_, CH_4_, and N_2_O, in three consecutive seasons from August 2019 until September 2021. The sampling interval for CO_2_ was once a week to every two weeks depending on the season. The sampling for N_2_O and CH_4_ was less frequent, especially in the year 2021. Permanent gas sampling spots were established in both vine rows and driving lanes by inserting PVC collars of 20 cm diameter and 11 cm height as chamber anchors with an insertion depth of about 7-8 cm. Each of the three treatments (Co, GC, and BCS with two repetitions each) contained a total of eight collars (i.e., at 3 slope positions in the driving lanes, and at 5 slope positions in the vine rows). The aboveground height of each collar was recorded each time for total chamber headspace calculation. Maintenance for tightness and replacement in case of damage was done regularly to ensure good measurement quality. The area inside the collars was kept free of vegetation to avoid the influence of photosynthesis and root respiration on CO_2_ measurements. The measurements at each sampling day were taken sequentially from one sampling row after another, starting from mid-morning until late afternoon, covering all 48 sampling spots. The overall flux average for each day was calculated from the 16 measurement replicates, sampled at different times of the day, which helped to minimize the bias of the diurnal effect on total GHG flux estimates.

Two infrared gas analyzers, a portable Fourier Transform Infrared (FTIR) analyzer (DX4015, Gasmet Technologies, Vantaa, Finland) for combined CO_2_, CH_4_, and N_2_O analysis, and a portable IR analyzer (LI-8100A analyzer, LI-COR Biosciences, Lincoln, NE, USA) for CO_2_ analysis, were used for gas flux measurement. A 20 cm survey chamber (8100-103, LI-COR Biosciences) with a factory-made pressure equilibration vent was used with the analyzers for sampling. Multiple pretests in the laboratory showed a very good agreement of CO_2_ flux estimates between the analyzers.

#### 2.3.1 Flux calculation

The emission of GHG from the soil surface was determined by linear increase of gas concentration in the chamber headspace over time (Parkin and Venterea, 2010; Collier et al., 2014). During each flux calculation of CO_2_ analysis with the LI-COR analyzer, the first 30 s of the concentration curve were discarded to account for the initial disturbance caused by chamber deployment and the time required to achieve a steady headspace mixing. In the case of the FTIR, the measurement of the first minute for all gas species was discarded for the same reason (Davidson et al., 2002; Görres et al., 2015; Jordan et al., 2016; Zhao et al., 2018). The slope of the concentration versus time was accepted if R^2^ > 0.8. The flux of CO_2_, CH_4_, and N_2_O was determined by the following formula (LI‐COR, 2010):


$$F_{C}\;=\;\frac{dC}{dt}\;\cdot \;\frac{10VP_{0}}{RA\left(T_{0}\;+\;273.15\right)}$$

where Fc is the flux (in μmol m^−2^ s^−1^ or nmol m^−2^ s^−1^), dC/dt is the initial rate of change in water-corrected mole fraction of gas species (µmol mol^-1^ or nmol mol^-1^) inside the chamber (slope of the linear regression), V (cm^3^) is the total volume of the closed loop (i.e., chamber, tubing, analyzer volume), A (cm^2^) is the soil surface area, P_0_ (kPa) is the initial pressure, T_0_ (°C) is the initial air temperature and R (= 8.314 L kPa K^−1^ mol^−1^) is the ideal gas constant. The unit was converted to mg m^-2^ h^-1^ or μg m^-2^ h^-1^ by multiplying the molecular weight of C and N of the respective gas species.

Total C loss in the form of CO_2_ over the two years of sampling was calculated as cumulative CO_2_ flux using the trapezoidal integration method, which is based on approximating the area under the curve of measured CO_2_ flux values versus time by dividing it into a series of trapezoids and summing the areas of each trapezoid. The area of each trapezoid was calculated by multiplying the average CO_2_ emission rate of two neighboring time points of CO_2_ emission measurements by the time in hours between these two time points (Singh et al., 1999).

### 2.4 Plant phenotyping

Plant phenotyping was performed over four consecutive years (2019-2022) using the same set of 21 grapevines per vine row in every season leading to a total of 84 investigated plants for each of the three experimental treatments, i.e. BCS, GC, and Co.

#### 2.4.1 Visual assessments

In all four years, the important phenological stages veraison (BBCH 83) and harvest (BBCH 89) (Lorenz et al., 1995) were assessed in the field as day of the year (DOY). From 2020-2022, bud break (BBCH 9) and full flowering (BBCH 65) (Lorenz et al., 1995) were considered as well.

In 2021, frequent rain events occurred, which lead to natural infestations of the grape bunches with *Botrytis* bunch rot. The amount of infested bunches per vine (incidence rate) and the severity per bunch (incidence level) were rated following a 6-class and 5-class classification scheme, respectively. Incidence rate: class 0: none, class 1: 1 bunch, class 3: < 50% of bunches, class 5: 50% of bunches, class 7: > 50% of bunches, class 9: all. Incidence level: class 1: single berries (< 5%), class 3: one small infection area, class 5: several small infection areas, class 7: large infection area, class 9: whole bunch infected.

#### 2.4.2 Leaf chlorophyll and nitrogen content

Leaf chlorophyll content (LCC) and nitrogen balance index (NBI) were measured using the Dualex 4 Scientific sensor (Force-A, Orsay, France). For this purpose, three mature leaves per plant were selected from different shoots above the grape berry zone in the middle of the canopy and each leaf was measured from the adaxial side on three interveinal positions. Throughout the four experimental years, sensor data acquisition was performed at three phenological time points: berries pea-sized (BBCH 75), veraison (BBCH 83), and harvest (BBCH 89) (Lorenz et al., 1995).

#### 2.4.3 Leaf macronutrient content

Leaf macronutrient content of 12 representative grapevines per treatment was analyzed in 2019, 2020, and 2021. Two weeks after veraison three mature leaves were collected of each vine, rinsed with ddH_2_O in the lab, freeze dried, and ground individually into powder. Approximately 300 mg of ground leaf powder were digested with 8 mL nitric acid (HNO_3_) (65%) in a microwave accelerated reaction system (Mars One^®^, CEM Corporation, Matthews, NC, USA). Samples were then transferred to glass flasks and ddH_2_O was added to reach a volume of 50 mL. Before macronutrient content analysis, samples were filtrated and diluted with three parts of cesium chloride (CsCl) solution (1 g/L). Magnesium (Mg), potassium (K), and calcium (Ca) content was analyzed by atomic absorption spectroscopy (240 FS AA, Agilent Technologies Inc., Santa Clara, CA, USA) using an acetylene/air oxidizing flame and a multielement lamp for detection. The system was equipped with an autosampler (SPS 3, Agilent Technologies Inc., Santa Clara, CA, USA) and data acquisition as well as processing was conducted using the corresponding software SpectrAA (Agilent Technologies Inc., Santa Clara, CA, USA). All measurements were automatically performed in triplicates.

#### 2.4.4 Berry impedance

Berry impedance measurements provide information about the cuticle and its waxy layers. Relative impedance (Z_REL_) of single berries was determined in all four experimental years close to harvest when berries reached on average 17.1-18.3°Brix. Per experimental treatment, 560 visually intact and unharmed berries were detached in the field by cutting their pedicels without touching the berry surface. Measurements were performed using a BI sensor with an intern resistance of two times 18 kOhm according to the detailed protocol of Herzog et al. (2015). Each berry was measured once at a randomly chosen spot close to the pedicel and, afterwards, sugar content of the berries was determined with a digital refractometer (VWR International, Radnor, PA, USA). For reference, 15 further cultivars and genotypes (**Supplementary Table 1**) were analyzed each year with a sample size of 20 berries.

#### 2.4.5 Texture analysis

In 2021 and 2022, texture was analyzed of a subset of 160 berries per experimental treatment directly after impedance measurements. Each berry was measured once at a randomly chosen spot on the lateral side as described by Letaief et al. (2008). For this purpose, a texture analyzer TA.XT Express equipped with a needle probe (P/2N, ø at tip 0.3 mm) (Stable Micro Systems, Godalming, UK) was used and data acquisition as well as analysis was performed with the software Exponent Lite Express (Stable Micro Systems, Godalming, UK). Texture analysis provides information about different berry skin characteristics: berry skin resistance described by maximum force (TA_FORCE_), berry firmness described by area (TA_AREA_), and berry skin elasticity described by gradient (TA_GRAD_) (Herzog et al., 2022).

#### 2.4.6 3D bunch architecture

During harvest 2021 and 2022, 24 representative grape bunches per experimental treatment were collected and brought to the laboratory, where 360° 3D point clouds of these bunches were generated using Artec Spider 3D scanner (Artec 3D, L-1466, Luxembourg) according to Rist et al. (2018). Analysis of these point clouds was performed automatically with the 3D Bunch Tool that was described by Rist et al. (2018). Thereby, different grape parameters can be determined: berry number per bunch (BN), mean berry diameter (MBD), mean berry volume (MBV), total berry volume (TBV), convex hull volume of a bunch (BC), bunch width (BW), and bunch length (BL).

#### 2.4.7 Grape quality parameters

Total soluble solids (TSS) expressed as glucose and fructose content, pH value, titratable acidity, and yeast-assimilable nitrogen (YAN) were determined as grape quality parameters at harvest in all four experimental years. Pooled samples of three grapevines were collected leading to a total of 28 samples per experimental treatment. The berries were mechanically homogenized and the juice without skins was then analyzed by Fourier-Transform Infrared spectroscopy (FTIR) (WineScan SO 2, FOSS Analytical A/S, Hillerød, Denmark). All measurements were automatically performed in duplicates.

### 2.5 Statistical analysis

Statistical analyses were performed using R version 4.1.2 (R Core Team, 2020) with ggplot2 package (Wickham, 2016) for visualization. For analysis of GHG emissions, data was tested for normal distribution and homogeneity of variance with the Shapiro-Wilk test and Levene test, respectively, before selecting a suitable statistical method for treatment comparison. In the case of non-normal distribution of data, log transformation was performed. A linear mixed-effect model was applied to test the effects of variables on CO_2_ flux. Treatments, seasons, and years were considered fixed effects, while replication was set as random. In terms of N_2_O, a constant value of 1 to the entire flux data was added due to the presence of a large number of non-detectable fluxes (62.84%), and a linear mixed effect model was performed subsequently (Jordan et al., 2016). The differences in cumulative flux estimates of the treatments were analyzed with one-way analysis of variance (ANOVA). Test of significant differences between individual treatment groups was performed by Tukey’s Honest Significant Difference (HSD) *post-hoc* comparison for balanced data and Tukey-Kramer approximation for unbalanced data.

A similar approach was followed for the analysis of plant phenotyping data with some modifications. If data were not normally distributed either log or quadratic transformation was performed. Linear mixed-effect models using the lme4 package (Bates et al., 2015) were applied with experimental year, treatment, and the interaction of both as fixed effects and individual grapevines as random effects. Modelling was followed by ANOVA and Tukey’s HSD test (p < 0.05) for which emmeans package (Lenth, 2022) was implemented.

## 3 Results

### 3.1 Greenhouse gas measurements

**Figure 2:**
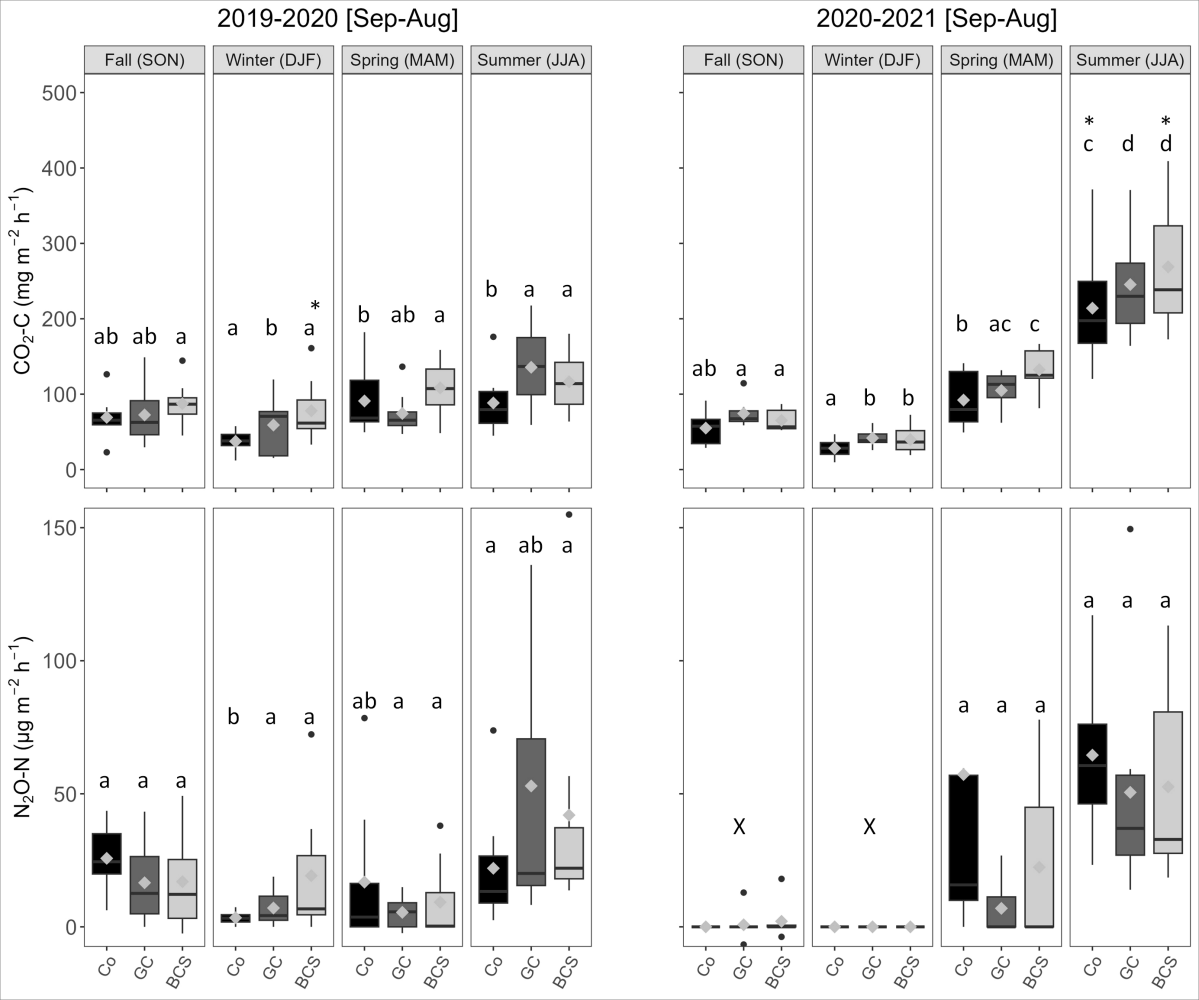
Seasonal average emissions of CO_2_-C (top) and N_2_O-N (bottom) for different treatments (Co, GC, and BCS) over two sampling years: 2019-2020 [Sep-Aug] and 2020-2021 [Sep-Aug]. Mean values are represented by gray diamond symbols within each box. Different letters denote significant differences among individual treatments across seasons. Differences between treatments within each season were not significant, hence not indicated. The “X” symbol inside the graph marks data points excluded from statistical analysis due to insufficient sample size and high amount of undetectable values. The asterisk (*) mark inside the graph indicates a significantly higher average flux of a treatment between years in a particular season.

Among the measured greenhouse gases, only CO_2_ showed regular emission and a dynamic pattern in relation to the changing meteorological condition. Although not statistically significant (p > 0.05), the overall emission of CO_2_ was higher in the treatments than in the control in most of the sampling period (**Figure 2**). The average CO_2_ emissions in the first sampling year, 2019-2020 [Sep-Aug], remained at a moderate level, ranging from the lowest (58.5 ± 4.3 mg m^-2^ h^-1^) in winter (DJF) to the highest (113.6 ± 3.3 mg m^-2^ h^-1^) in summer (JJA), regardless of treatments, due to cold or dry conditions. In the second sampling year, 2020-2021 [Sep-Aug], the emissions followed a similar pattern, with the lowest average emission of 36.6 ± 2.8 mg m^-2^ h^-1^ in winter (DJF) and the highest average emission of 244.5 ± 12.8 mg m^-2^ h^-1^ in summer (JJA). The average emissions from Co, GC, and BCS treatments in JJA of the first year were 88.6 ± 4.9, 136.1 ± 6.8, and 116.2 ± 4.9 mg m^-2^ h^-1^, respectively, while in the second year, they were 214.3 ± 19.9, 247.4 ± 21.8, and 271.3 ± 24.4 mg m^-2^ h^-1^, respectively. The significantly higher emission in JJA of the second year compared to the first year was attributed to heavy rainfall during that period. The total estimated C loss from Co, GC, and BCS treatments over the two years of sampling was 15.19, 18.26, and 20.54 Mg h^-1^, respectively.

The temporal dynamics of N_2_O in the field included both net emission (production) and net uptake (consumption) of N_2_O. On many of the sampling days and positions, no detectable flux could be found. Non-detectable flux estimates were included in the analysis as zero values in order to avoid overestimation of flux averages (Jordan et al., 2016). There was no significant (p > 0.05) treatment effect on N_2_O emission throughout the whole sampling period (**Figure 2**). Similar to CO_2_, higher N_2_O emissions were observed in JJA compared to other seasons in both years. The average emission of N_2_O in the first year remained at a very low to moderate level, ranging from 9.9 ± 2.1 µg m^-2^ h^-1^ in DJF to 38.9 ± 9.7 µg m^-2^ h^-1^ in JJA, regardless of treatments. In the second year, N_2_O sample collection in SON and DJF was limited by COVID-19-related restrictions, and the few collected samples contained undetected fluxes, which were excluded from the statistical analysis and comparison. The average emission in JJA of the second year was 55.9 ± 7.9 µg m^-2^ h^-1^, influenced by the high precipitation in June.

Although little production and consumption of CH_4_ was detected at isolated points, the overall contribution of CH_4_ to the total GHG flux estimate was negligible.

### 3.2 Plant phenotyping

#### 3.2.1 Visual assessments

Throughout the investigated growing seasons, four important phenological stages were assessed as mean DOY for each of the experimental treatments (**Table 2**). Clear differences can be observed between the different years with phenological stages of bud break (BBCH 9), full flowering (BBCH 65), and veraison (BBCH 83) being reached earliest in 2020 and latest in 2021 and the years 2019 and 2022 showing intermediate dates. Time point of harvest (BBCH 89) deviated slightly from this pattern with again latest DOY in 2021, but earliest DOY in 2022. Between the experimental variants, however, no differences could be observed since they reached the respective phenological stages on average on the same DOY.

**Table 2:** Phenological stages of bud break (BBCH 9), full flowering (BBCH 65), veraison (BBCH 83), and harvest (BBCH 89) expressed as mean day of the year for vines grown on biochar-compost substrate (BCS), on greenwaste compost (GC), or in control (Co) rows for the years 2019 – 2022.

	BBCH 9			BBCH 65			BBCH 83			BBCH 89		
	BCS	GC	Co	BCS	GC	Co	BCS	GC	Co	BCS	GC	Co
**2019**	na	na	na	na	na	na	225	225	225	253	253	253
**2020**	99	99	99	154	154	154	214	214	214	258	258	258
**2021**	116	116	116	170	170	170	235	235	235	265	265	265
**2022**	110	110	110	157	157	157	217	217	217	250	250	250

na, not assessed.

**Figure 3:**
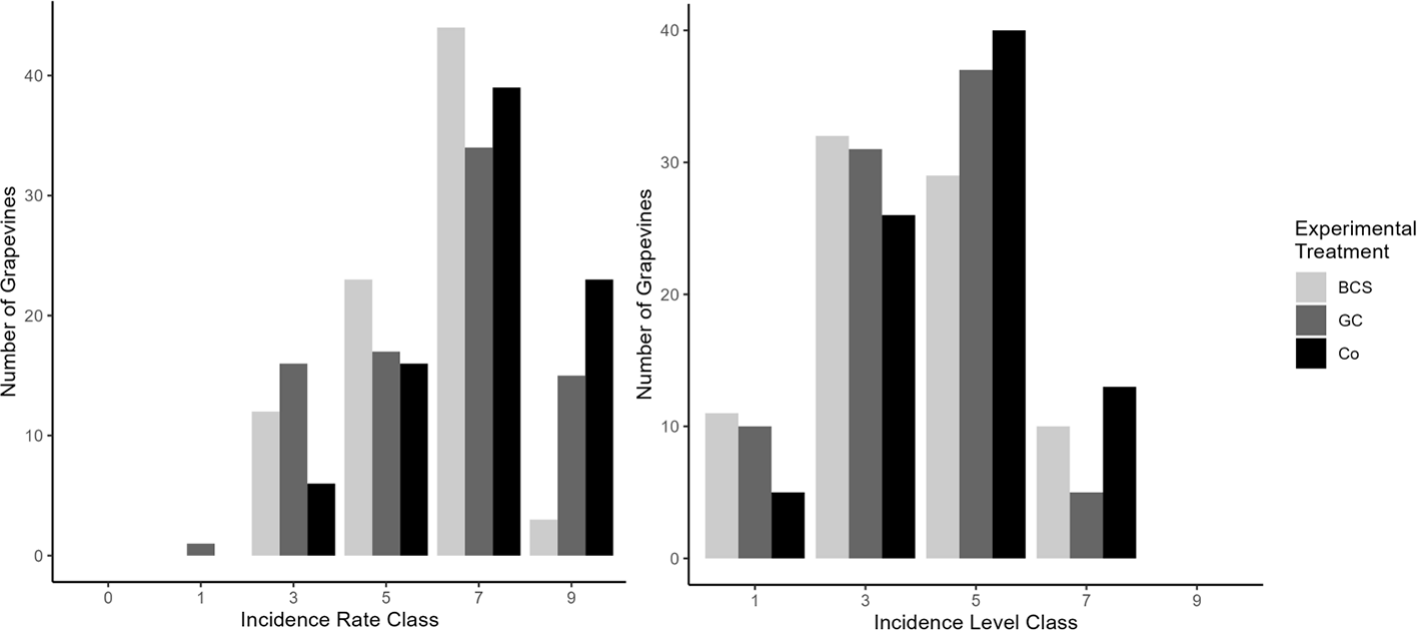
Incidence rate (left) and incidence level (right) of *Botrytis* bunch rot in the field in 2021 for vines grown on biochar-compost substrate (BCS), greenwaste compost (GC), or in control (Co) rows. Arrows indicate median of the respective group.

Natural *B. cinerea* infestation could be observed in the field only in 2021. In general, all vines were affected by *Botrytis* bunch rot (**Figure 3**) since no observations of incidence rate class 0 (=no infested bunches) were made. For all experimental treatments, the incidence rate was predominantly class 7 meaning that more than 50% of all bunches showed symptoms of *Botrytis* bunch rot. However, for plants grown on BCS a tendency towards fewer infected bunches became apparent with fewer individuals in class 9. The incidence level showed a similar pattern for all three experimental treatments with most individuals in classes 3 and 5.

#### 3.2.2 Leaf chlorophyll and nitrogen content

LCC mean values differed significantly between the four years examined with lowest LCC in 2020 and highest LCC in 2019 (**Table 3**). Vines grown in control rows showed in general lower LCC than vines grown on the two OA. Significant differences could be observed between control vines and vines grown on BCS in the years 2019 and 2020 as well as between control vines and vines grown on GC in 2020 and 2021. Between the two OA, however, no significant differences could be observed and, in 2022, no differences in LCC became apparent between the three treatments.

**Table 3:** Mean leaf chlorophyll content (LCC) [µg/cm^2^] and mean nitrogen balance index (NBI) for vines grown on biochar-compost substrate (BCS), on greenwaste compost (GC), or in control (Co) rows for the years 2019 – 2022.

	LCC			NBI		
	BCS	GC	Co	BCS	GC	Co
**2019**	29.3 ^Cab^	29.6 ^Cb^	28.8 ^Da^	14.6 ^Bab^	14.7 ^Bb^	14.2 ^Ca^
**2020**	24.5 ^Ab^	24.9 ^Ab^	22.4 ^Aa^	11.6 ^Ab^	11.9 ^Ab^	10.6 ^Aa^
**2021**	25.2 ^Aab^	25.5 ^ABb^	24.6 ^Ba^	11.6 ^Aa^	11.7 ^Aa^	11.5 ^Ba^
**2022**	26.7 ^Ba^	26.2 ^Ba^	26.2 ^Ca^	14.6 ^Ba^	14.5 ^Ba^	14.2 ^Ca^

Different lower case letters indicate significant difference between treatments within the same season, while different capital letters indicate significant differences between the same treatments at different seasons according to Tukey’s HSD (p < 0.05).

For mean NBI values (**Table 3**), vines exhibited lowest NBI in 2020 and highest NBI in 2019 and 2022. Similar to LCC, vines grown in control rows exhibited lower NBI values in comparison to vines grown on BCS or GC. Again, no differences could be observed in NBI for vines grown on the two OA. In 2019, control plants showed significant differences only towards vines grown on GC and, in 2020, to vines grown on either BCS or GC. However, in 2021 and 2022 no differences between the treatments could be observed.

#### 3.2.3 Leaf macronutrient content

Leaf content of both Mg and K was lowest in 2021 (**Table 4**) and showed no differences between the years 2019 and 2020 for BCS and Co. For GC, however, Mg contents were higher in 2020 than 2019 and K contents were lower in 2020 than 2019. Variations in leaf Ca content between the years were only apparent for plants grown on GC with higher values in 2020 than 2019. No measurable effect of BCS or GC could be detected in comparison to control plants.

**Table 4:** Mean leaf content of magnesium (Mg) [mg/L], potassium (K) [mg/L] and calcium (Ca) [mg/L] for vines grown on biochar-compost substrate (BCS), on greenwaste compost (GC), or in control (Co) rows for the years 2019 – 2021.

	Mg			K			Ca		
	BCS	GC	Co	BCS	GC	Co	BCS	GC	Co
**2019**	12.6 ^Ba^	13.5 ^Ba^	13.2 ^Ba^	48.6 ^Ba^	51.6 ^Ca^	49.3 ^Ba^	169.1 ^Aa^	157.4 ^Aa^	164.9 ^Aa^
**2020**	13.3 ^Ba^	15.0 ^Ca^	13.5 ^Ba^	50.7 ^Ba^	46.9 ^Ba^	45.8 ^Ba^	168.3 ^Aa^	169.5 ^Ba^	171.1 ^Aa^
**2021**	10.6 ^Aa^	11.7 ^Aa^	11.8 ^Aa^	41.9 ^Aa^	38.6 ^Aa^	41.9 ^Aa^	163.7 ^Aa^	167.9 ^ABa^	166.9 ^Aa^

Different lower case letters indicate significant difference between treatments within the same season, while different capital letters indicate significant differences between the same treatments at different seasons according to Tukey’s HSD (p < 0.05).

#### 3.2.4 Berry impedance

**Figure 4:**
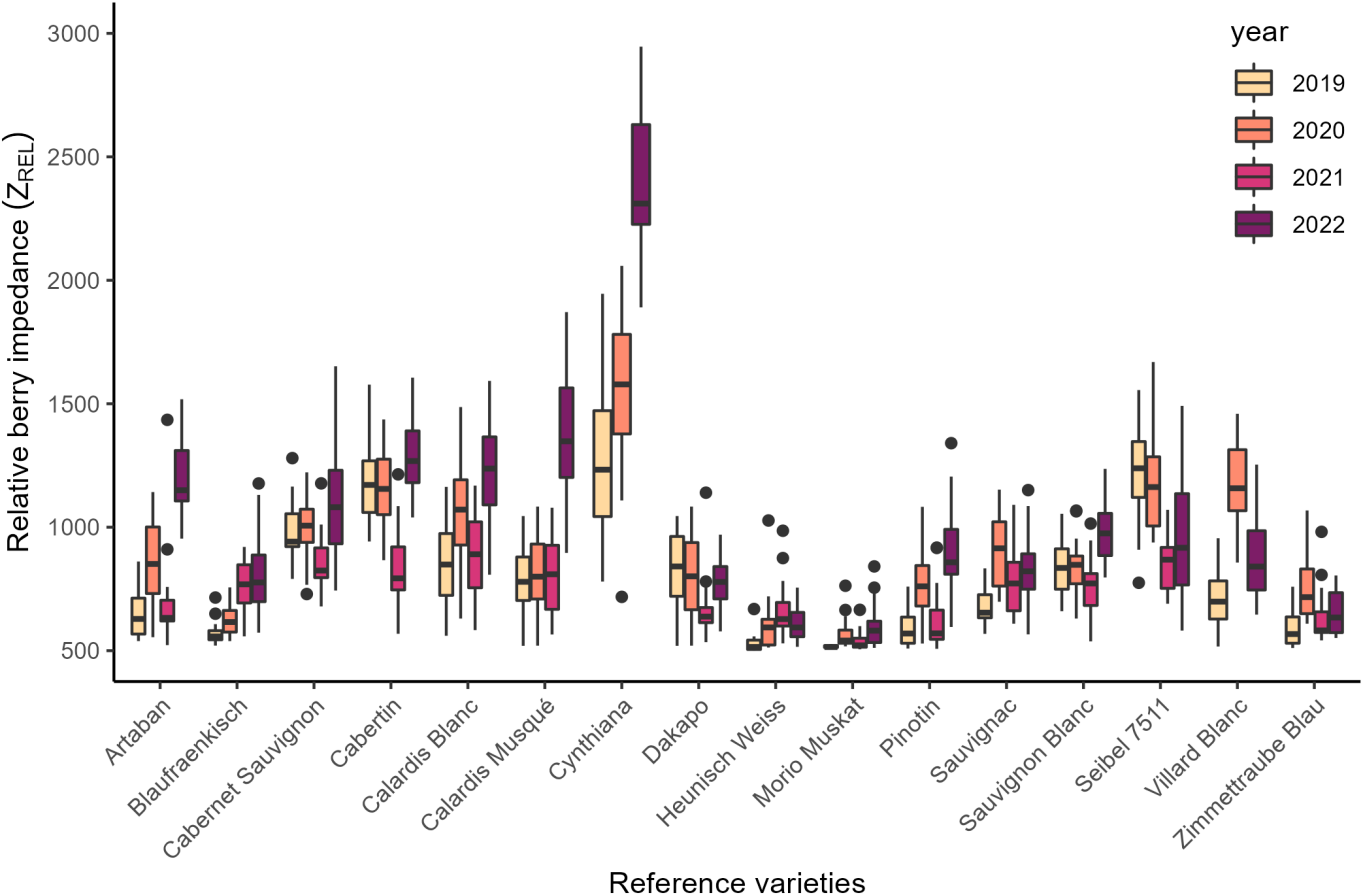
Relative berry impedance (Z_REL_) values of reference varieties for the years 2019-2022. N=20 berries per variety and year.

In general, medium Z_REL_ values can be expected of grapevine cultivar ‘Calardis Musqué’ in comparison to 15 reference varieties as is depicted in **Figure 4** indicating that the resilience towards *Botrytis* bunch rot is neither particularly high nor low.

Significant differences between Z_REL_ of the four experimental years became apparent with lowest values in 2021, followed by 2019 and 2020 and highest values in 2022 (**Table 5**). In fact, Z_REL_ in 2022 was almost twice as high as in 2021. Low Z_REL_ in 2021 could also be observed for reference varieties such as ‘Cabernet Sauvignon’, ‘Cabertin’, and ‘Dakapo’ as well as the strong increase in 2022 for ‘Artaban’, ‘Calardis Blanc’, ‘Cynthiana’, ‘Pinotin’, or ‘Sauvignon Blanc’ (**Figure 4**). In general, control vines had significantly higher Z_REL_ than plants grown on either BCS or GC in the years 2019, 2020, and 2022. In 2021, no significant differences between the experimental treatments could be observed.

**Table 5:** Mean relative berry impedance values (Z_REL_) for vines grown on biochar-compost substrate (BCS), on greenwaste compost (GC), or in control (Co) rows for the years 2019 – 2022.

	BCS	GC	Co
**2019**	866.5 ^Ba^	853.7 ^Ba^	984.4 ^Bb^
**2020**	1055.5 ^Cb^	985.9 ^Ca^	1179.5 ^Cc^
**2021**	836.7 ^Aa^	812.2 ^Aa^	833.2 ^Aa^
**2022**	1589.5 ^Da^	1584.9 ^Da^	1676.9 ^Db^

Different lower case letters indicate significant difference between treatments within the same season, while different capital letters indicate significant differences between the same treatments at different seasons according to Tukey’s HSD (p < 0.05).

#### 3.2.5 Texture analysis

Texture analysis of single berries showed significantly higher values for TA_FORCE_ and TA_AREA_ in 2022 than in 2021 (**Table 6**) and the opposite pattern for TA_GRAD_ with higher values in 2021. Vines grown on BCS had significantly higher TA_AREA_ values than vines grown in control rows over the two years analyzed. For both TA_FORCE_ and TA_GRAD_ no measurable effect of BCS or GC could be detected in comparison to Co.

**Table 6:** Mean values of berry skin resistance (TA_FORCE_) [N], berry firmness (TA_AREA_) [N·sec], and berry skin elasticity (TA_GRAD_) [N/sec] for vines grown on biochar-compost substrate (BCS), on greenwaste compost (GC), or in control (Co) rows for the years 2021 – 2022.

	TA_FORCE_			TA_AREA_			TA_GRAD_		
	BCS	GC	Co	BCS	GC	Co	BCS	GC	Co
**2021**	0.72 ^Aa^	0.70 ^Aa^	0.68 ^Aa^	0.46 ^Ab^	0.45 ^Aab^	0.42 ^Aa^	0.53 ^Ba^	0.52 ^Ba^	0.52 ^Ba^
**2022**	0.82 ^Ba^	0.81 ^Ba^	0.82 ^Ba^	0.58 ^Bb^	0.56 ^Bab^	0.55 ^Ba^	0.49 ^Aa^	0.48 ^Aa^	0.49 ^Aa^

Different lower case letters indicate significant difference between treatments within the same season, while different capital letters indicate significant differences between the same treatments at different seasons according to Tukey’s HSD (p < 0.05).

#### 3.2.6 3D bunch architecture

For all parameters analyzed in acquired 3D scans, significant differences between the two experimental years could be observed (**Table 7**). In 2021, grape bunches were larger with more and bigger berries than in 2022. However, no measurable effect of the two OA could be detected in comparison to control plants.

Table 7Mean values of berry number (BN), mean berry diameter (MBD) [mm], mean berry volume (MBV) [mL], total berry volume (TBV) [mL], convex hull volume of a bunch (BC) [mL], bunch width (BW) [mm], and bunch length (BL) [mm] for vines grown on biochar-compost substrate (BCS), on greenwaste compost (GC), or in control (Co) rows for the years 2021 – 2022.

**Table:** 

	BN			MBD			MBV			TBV		
	BCS	GC	Co	BCS	GC	Co	BCS	GC	Co	BCS	GC	Co
**2021**	175 ^Ba^	182 ^Ba^	178 ^Ba^	11.8 ^Ba^	11.8 ^Ba^	11.9 ^Ba^	915.9 ^Ba^	921.1 ^Ba^	930.1 ^Ba^	158.3 ^Ba^	165.1 ^Ba^	164.5 ^Ba^
**2022**	153 ^Aa^	148 ^Aa^	151 ^Aa^	11.4 ^Aa^	11.0 ^Aa^	11.1 ^Aa^	828.9 ^Aa^	755.2 ^Aa^	753.6 ^Aa^	120.7 ^Aa^	110.7 ^Aa^	109.8 ^Aa^

	BC			BW			BL		
	BCS	GC	Co	BCS	GC	Co	BCS	GC	Co
**2021**	641.4 ^Ba^	638.8 ^Ba^	630.7 ^Ba^	114.2 ^Ba^	115.7 ^Ba^	119.9 ^Ba^	139.6 ^Ba^	140.8 ^Ba^	140.6 ^Ba^
**2022**	449.0 ^Aa^	417.4 ^Aa^	414.6 ^Aa^	99.9 ^Aa^	94.6 ^Aa^	95.2 ^Aa^	125.7 ^Aa^	124.9 ^Aa^	127.8 ^Aa^

Different lower case letters indicate significant difference between treatments within the same season, while different capital letters indicate significant differences between the same treatments at different seasons according to Tukey’s HSD (p < 0.05).

#### 3.2.7 Grape quality parameters

Glucose and fructose content of collected berries was higher in 2019 and 2020 than in 2021 and 2022 (**Table 8**). pH values were lowest in 2022 and highest in 2020 with 2019 and 2021 showing comparable intermediate results. Titratable acidity and YAN differed in all four experimental years with lowest amounts in 2019 and highest amounts in 2021. When regarding the three experimental treatments, no differences could be observed for glucose, titratable acidity, and YAN contents. In all four years, pH values were lowest in berries of control vines in comparison to berries from BCS or GC. Fructose content in berries differed only in the year 2020 with Co having significantly lower content than GC.

**Table 8:** Mean values of glucose [g/L], fructose [g/L], pH, titratable acidity (TA) [g/L], and yeast assimilable nitrogen (YAN) [mg/L] for vines grown on biochar-compost substrate (BCS), on greenwaste compost (GC), or in control (Co) rows for the years 2019 – 2022.

	Glucose			Fructose			pH			TA			YAN		
	BCS	GC	Co	BCS	GC	Co	BCS	GC	Co	BCS	GC	Co	BCS	GC	Co
**2019**	97.5 ^Ba^	95.5 ^Ba^	90.5 ^Ba^	109.2 ^Ba^	106.8 ^Ba^	102.3 ^Ba^	3.36 ^Bb^	3.34 ^Bb^	3.29 ^Ba^	6.26 ^Aa^	6.66 ^Aa^	6.96 ^Aa^	37.1 ^Aa^	41.6 ^Aa^	47.4 ^Aa^
**2020**	91.5 ^Ba^	94.9 ^Ba^	89.7 ^Ba^	106.3 ^Bab^	110.2 ^Bb^	104.3 ^Ba^	3.50 ^Cb^	3.50 ^Cb^	3.40 ^Ca^	7.09 ^Ba^	7.19 ^Ba^	7.71 ^Ba^	101.9 ^Ca^	108.0 ^Ca^	79.0 ^Ca^
**2021**	80.4 ^Aa^	78.3 ^Aa^	82.1 ^Aa^	91.5 ^Aa^	89.2 ^Aa^	93.1 ^Aa^	3.35 ^Bb^	3.34 ^Bb^	3.35 ^Ba^	9.39 ^Da^	9.56 ^Da^	9.17 ^Da^	141.2 ^Da^	138.4 ^Da^	126.9 ^Da^
**2022**	79.8 ^Aa^	81.7 ^Aa^	79.7 ^Aa^	90.5 ^Aa^	92.0 ^Aa^	90.9 ^Aa^	3.29 ^Ab^	3.29 ^Ab^	3.25 ^Aa^	7.92 ^Ca^	8.02 ^Ca^	8.07 ^Ca^	71.2 ^Ba^	74.8 ^Ba^	71.5 ^Ba^

Different lower case letters indicate significant difference between treatments within the same season, while different capital letters indicate significant differences between the same treatments at different seasons according to Tukey’s HSD (p < 0.05).

## 4 Discussion

Against the background of an envisaged C sequestration, a biochar-compost substrate and a greenwaste compost were incorporated to greater soil depth than in common practice (i.e. into the subsoil of an experimental vineyard) before planting. A rapid turnover of these organic amendments would lead to higher GHG emissions, which in turn would be contradictory to the aim of climate change mitigation. In order to estimate the degree of decomposition of the organic materials, the amount of emitted GHG was evaluated for two consecutive years after the first year of incorporation. Similar to other studies, the seasonal variation of GHG emission in the investigated vineyard was largely driven by the dynamics of temperature and moisture conditions (Rey et al., 2005; Carlisle et al., 2006; Steenwerth et al., 2010; Vicca et al., 2014; Yu et al., 2017). Thereby, the rates of CO_2_ and N_2_O emission were comparable with other studies conducted in vineyard soils in different climates (Carlisle et al., 2006; Steenwerth et al., 2010; Horel et al., 2018; Marín-Martínez et al., 2021; Minardi et al., 2022). The observed increase in CO_2_ and N_2_O emission in 2021 could be related to a rapid increase in biological activity that is likely to occur after rewetting events (**Figure 1**) (Franzluebbers et al., 2000; Rey et al., 2005; Steenwerth et al., 2010; Laffely et al., 2020; Jiang et al., 2021).

The increased CO_2_ emissions in amended soil throughout the whole measurement period may be attributed to the gradual decomposition of the applied OA in deeper soil layers over an extended period. Since the stability of C in the subsoil is predominantly limited by the physical separation of the decomposer and the substrate (Salomé et al., 2010), air intrusion to greater depths – enabled by deep shrinkage cracks in the vertic soils of our experimental vineyard during dry periods – may have favored decomposition processes. The increased CO_2_ emissions in the course of the experimental period are in contrast to previous works that reported higher emissions of CO_2_ in the first few months to one year after OA incorporation (Major et al., 2010; Bass et al., 2016). Treatment of soil with OA is often associated with higher CO_2_ emissions due to the introduction of labile compounds for microbial degradation (Rogovska et al., 2011; Mukherjee and Lal, 2013; Thangarajan et al., 2013; Agegnehu et al., 2016; Bass et al., 2016) which applies to GC, but also to BCS, as both contain compost. However, despite a slight tendency to larger CO_2_ emissions in OA treatments, we did not find significant differences from the control. This could be mainly due to the large spatial variability of soils and, in consequence, in our data.

In agreement with similar works in vineyards, we could not detect any negative, i.e. stimulatory, effect of organic treatments on N_2_O dynamics (Horel et al., 2018; Marín-Martínez et al., 2021). In our work, the GC and BCS treatment had slightly lower N_2_O emissions at the end of the experiment than the control indicating the potential of OA for N_2_O mitigation. Other studies have emphasized the contribution of high-carbon OA on reducing N_2_O emission by enhancing microbial immobilization of the available substrate (Reichel et al., 2018) and by sorption of toxic chemicals that inhibit microbial growth (Gundale and DeLuca, 2007). The positive effect of organic material addition could also include reduced bulk density and increased aeration in the soil that minimizes N_2_O producing conditions (i.e. denitrification) (Mylavarapu and Zinati, 2009; Case et al., 2012).

CH_4_ production and consumption at our study site were negligible, with very low detectable flux at scattered points. CH_4_ production occurs primarily under anaerobic conditions by methanogenic archaea in the presence of an organic C source. However, vineyards are not considered a CH_4_ source (Carlisle et al., 2010) and if they are up to 90% of the produced methane can be oxidized to CO_2_ by methanotrophic bacteria in the presence of oxygen (Le Mer and Roger, 2001; Serrano-Silva et al., 2014).

Incorporation of OA could not only affect GHG emissions, but also grapevines since soil properties and the incorporation of different organic materials have been shown to substantially influence different plant parameters (White et al., 2007; Morlat, 2008; van Leeuwen et al., 2018). In the four years of our study, we assessed the influence of a deep incorporation on a wide range of different leaf and berry traits mostly using sensor-based approaches, but only few of these traits were significantly affected by the OA. Note that the soils of the experimental vineyard revealed silty-loamy to loamy-clayey texture, resulting in a rather large water holding capacity. With respect to the literature reviewed by Blanco-Canqui (2021), the amounts of OA here were too small to significantly improve soil physical properties. Therefore, water supply to the vines was dominated by slope position and meteorological conditions rather than by OA treatments.

In all four seasons, grapevines of the three experimental treatments reached phenological stages on average on the same day of the year indicating that grapevine development was not affected by OA. Mondini et al. (2018) reported only a tendency of vine shoot compost to delay berry maturation, but did not consider further phenological time points. Other studies focused on vegetative growth for the assessment of grapevine development and found contradictory results. While Gaiotti et al. (2017) and Tarricone et al. (2018) reported increases in leaf area and root system caused by soil amendments, Mugnai et al. (2012) found no effects on phenological parameters.

Both LCC and NBI were significantly increased in plants grown on either of the two OA in the first few years after incorporation. Compost application to vineyards has been shown to improve total soil N content, in general, and plant available N, in particular (Morlat and Chaussod, 2008), which explains the increased leaf N contents we observed and that have also been demonstrated by Wilson et al. (2021) and Schmidt et al. (2014). However, significant effects were only of transient nature as they decreased with time. This might be caused by GC and BCS being incorporated as a single dose before planting due to regulations by the German Fertilization Ordinance (DüV), the wide C:N ratio of the substrates, and their deep incorporation what may have led to a reduced subsequent redelivery of N by the substrate. This theory is in line with several other publications that reported significant differences in LCC and N content only in plants with high substrate applications (Morlat, 2008; Tarricone et al., 2018). Furthermore, Wilson et al. (2021) tested different compost application rates and found a single application at low rates to have little or no detectable effects on vine balance or juice characteristics. Moreover, Schmidt et al. (2014) also used only moderate amounts of compost as well as biochar amendments and observed significant differences in leaf N content only in the first year after incorporation. The increased LCC content observed in our study, however, cannot be correlated to an increased Mg content in leaves collected shortly after veraison since macronutrient content was generally not affected by GC and BCS indicating that nutrient supply was sufficient also in un-amended control rows. In a long-term experiment over 28 years, Morlat (2008) did also not detect any effects of organic amendments on leaf Mg content, thus, confirming our results. In contrast to our data, leaf K and Ca content has been found to be significantly increased by different substrate applications (Morlat, 2008; Tangolar et al., 2020; Wilson et al., 2021) what might be attributed to the grapevines’ age that ranged from 4-11 years while our study focused on newly planted vines. The vines’ root systems might therefore not have fully reached OA in the subsoil. However, in an excavation experiment performed in sandy soil Schmitz (2022) found the longest root of ‘Calardis Musqué’ plants four months after planting to have an average length of 105 cm and an especially high root area from a soil depth of 70 cm and deeper. Since OA were incorporated in up to 0.6 m depth and our assessments started in the year after planting, it can be expected that the root system was in contact with the OA. In order to gain more insights into root development and the influence of OA on different leaf traits, rhizotrons or potted plants could be used in future, allowing controlled experimental conditions with defined amounts of OA per plant.

When considering berry traits and grape quality parameters, our results showed only partial and not always significant effects of OA incorporation. Berry surface characteristics like the cuticle, epicuticular waxes, and skin thickness, or bunch architecture are known to be of high importance for the susceptibility or resilience towards different pest and diseases – especially *Botrytis* bunch rot (Herzog et al., 2015; Rist et al., 2018; Entling et al., 2019; Herzog et al., 2022). Of the assessed traits in this study, impedance differed significantly between the three experimental treatments (except in 2021) with berries grown in non-amended control rows having the highest values meaning that the berry cuticle and its waxy layers are thicker what could contribute to a higher resilience towards *Botrytis* bunch rot (Herzog et al., 2015; Herzog et al., 2022). Unfortunately, *Botrytis* infestations in the field occurred only in 2021 – the year with no significant differences in impedance between the experimental treatments –, therefore no conclusions could be drawn. In order to investigate the effect of amendment incorporation on the trait *Botrytis* resilience further, an artificial infection test in the laboratory might be a suitable approach. While berry firmness was lowest in control rows, other traits like berry skin resistance and elasticity as well as bunch architecture traits were not significantly affected by the OA. Bunch architecture analyses provide information, for instance, about berry number and size as well as whole bunch size, which are important characteristics for yield assessment (Rist et al., 2018). In line with our work, Gaiotti et al. (2017) and Mugnai et al. (2012) did not find production parameters to be influenced by compost application. Wilson et al. (2021), though, reported contrasting results with heavier berries and clusters after high doses of compost treatments. Furthermore, biochar application has also been found to lead to heavier berries and clusters (Genesio et al., 2015). Comparing results of different studies regarding yield, however, is generally rather difficult not only because of age and cultivar of investigated grapevines but also because of different origin, composition, and amount of amendments incorporated. The same accounts for grape quality parameters. Although many studies observed no differences in sugar content, TA, or pH after amendment incorporation (Mugnai et al., 2012; Gaiotti et al., 2017; Mondini et al., 2018; Botelho et al., 2021; Wilson et al., 2021), some confirmed our results of increased pH values in amended vine rows (Morlat and Symoneaux, 2008; Chan and Fahey, 2011; Schmidt et al., 2014). Despite the differences in pH we observed between the three experimental variants being significant, the fluctuations in pH between the four seasons examined were much stronger, which actually was the case for all investigated leaf and berry traits of this study. Several published works regarding the effects of OA on different grapevine parameters confirm our observations, that seasonal variations were in some cases much more pronounced than the influence of amendment incorporation on the assessed traits (Chan and Fahey, 2011; Mugnai et al., 2012; Schmidt et al., 2014; Genesio et al., 2015; Olego et al., 2022).

The year 2021 with a mean annual temperature of 10.4°C and total precipitation of 814.5 mm was significantly cooler and wetter than the other three experimental years (11.5-12.3°C, 630.4-656.5 mm). These contrasts became very clear when looking at phenological stages, which were reached several days later in 2021. Although differences were also obvious in leaf parameters, they were most pronounced in berry and grape traits what is in line with the fact that yield and quality – especially of non-irrigated vineyards – are highly dependent on meteorological conditions throughout the growing season (Ferrer et al, 2017, van Leeuwen et al., 2004). In contrast to the years 2019, 2020, and 2022, the year 2021 was characterized by low sugar contents and high acidity as can be expected by rather cool climate conditions (Ferrer et al., 2017; Venios et al., 2020). Differences in impedance were most pronounced between the years 2021 and 2022 and could also be observed in several reference cultivars. Low impedance values in 2021 might be due to many rain events throughout the growing season what might have led to a reduction of epicuticular waxy layers. This could also explain the lack of differences between the three experimental variants in this year. On the contrary, almost no rain events occurred during berry development in 2022 presumably leading to undisturbed waxy layers and consequently higher impedance values. Other berry surface traits as well as bunch architecture were assessed in 2021 and 2022 only. Due to the higher amount of precipitation in 2021, grapevines developed more and bigger berries leading to generally larger bunches. Furthermore, berry skin was more elastic but less resistant and overall berry firmness was lower in 2021 than 2022. The combination of the before mentioned trait differences and the increased water around harvest could be a suitable explanation for the occurrence of *Botrytis* bunch rot in the field in 2021 (Herzog et al., 2015; Rist et al., 2018; Tello and Ibañez, 2018; Dry et al., 2019; Herzog et al., 2022).

## 5 Conclusion

In the context of sustainable viticulture and climate change mitigation, deep incorporation of organic amendments in vineyard subsoils might be a suitable approach for long-term C storage. Therefore, in this work two OA – biochar-compost substrate and greenwaste compost – were incorporated in up to 60 cm depth of an experimental vineyard before it was planted with ‘Calardis Musqué’, a fungus-resistant cultivar, resulting in a drastic reduction of fungicide demand throughout the growing season and subsequently less tractor traffic in the vineyard, which further mitigates GHG emissions. Thus, fungus-resistant cultivars can make a valuable contribution to sustainable viticulture. In summary, our management approach had a limited effect on soil CO_2_ emissions and we did not observe a significant enhancement in non-CO_2_ greenhouse gas (N_2_O and CH_4_) emissions which would have run counter to the objectives of climate change mitigation. Deep incorporation induced no rapid turnover of the two OA. Differences in grapevine development as well as various leaf and berry traits between the two amendment treatments and the control were scarce and mostly of transient nature, which might be attributed to the limited amount of substrates incorporated due to regulations by the German Fertilization Ordinance (DüV). In general, seasonal variations were much more pronounced for both GHG emissions and plant traits than effects caused by the amendments. Since there were no obvious negative effects on the assessed parameters in this study, deep incorporation of either GC or BCS can be recommended. However, the results should be verified on further vineyards with varying soil properties. Finally, grapevines are perennial plants and soil parameters change rather slowly, thus, further studies are needed to investigate the mechanisms behind these effects and their potential impact on sustainable vineyard management in future.

## Data availability statement

The original contributions presented in the study are included in the article/**Supplementary Material**. Further inquiries can be directed to the corresponding author.

## Author contributions

NS: Conceptualization, Data curation, Formal Analysis, Investigation, Methodology, Validation, Visualization, Writing – original draft, Writing – review & editing. MI: Conceptualization, Data curation, Formal Analysis, Investigation, Methodology, Validation, Visualization, Writing – original draft, Writing – review & editing. RW: Conceptualization, Data curation, Formal Analysis, Investigation, Methodology, Validation, Writing – original draft, Writing – review & editing. SP: Conceptualization, Data curation, Formal Analysis, Funding acquisition, Investigation, Methodology, Project administration, Resources, Supervision, Validation, Writing – original draft, Writing – review & editing. NB: Conceptualization, Funding acquisition, Project administration, Resources, Supervision, Writing – review & editing. RT: Conceptualization, Funding acquisition, Project administration, Resources, Supervision, Writing – review & editing. KH: Conceptualization, Funding acquisition, Project administration, Resources, Supervision, Writing – review & editing.
